# Casparian strips prevent apoplastic diffusion of boric acid into root steles for excess B tolerance

**DOI:** 10.3389/fpls.2023.988419

**Published:** 2023-12-14

**Authors:** Keita Muro, Jio Kamiyo, Sheliang Wang, Niko Geldner, Junpei Takano

**Affiliations:** ^1^ Department of Agricultural Biology, Graduate School of Agriculture, Osaka Metropolitan University, Sakai, Japan; ^2^ Division of Applied Life Sciences, Graduate School of Life and Environmental Sciences, Osaka Prefecture University, Sakai, Japan; ^3^ Department Of Soil Science and Plant Nutrition, College of Resources and Environment, Huazhong Agricultural University, Wuhan, China; ^4^ Department of Plant Molecular Biology, University of Lausanne, Lausanne, Switzerland

**Keywords:** *Arabidopsis*, boron, toxicity, Casparian strip, apoplast, biosensor

## Abstract

Casparian strips are ring-like structures consisting of lignin, sealing the apoplastic space between endodermal cells. They are thought to have important functions in controlling radial transport of nutrients and toxic elements in roots. However, *Arabidopsis* mutants with a defective Casparian strip structure have been found to maintain nutrient homeostasis in ranges supportive of growth under standard laboratory conditions. In this study, we investigated the function of Casparian strips under excess boron (B) conditions using *sgn3* and *sgn4* mutants with defective Casparian strip development but which do not exhibit excessive deposition of suberin, another endodermal diffusion barrier. The growth of *sgn3* and *sgn4* mutants did not differ significantly from that of wild-type (WT) plants under different B conditions in plate cultures; however, they were highly sensitive to B excess in hydroponic culture, where transpiration drives the translocation of boric acid toward the shoot. In hydroponic culture with sufficient to excess boric acid, B accumulation in shoots of the *sgn3* and *sgn4* mutants was higher than that in the WT. A time-course tracer study using ^10^B-enriched boric acid at a sufficient or slightly excessive concentration showed higher translocation of B into shoots of the *sgn3* and *sgn4* mutants. Furthermore, a genetically encoded biosensor for boric acid expressed under a stele-specific promoter (*proCIF2:NIP5;1 5*′*UTR : Eluc-PEST*) visualized faster boric acid flux into the mutant steles. Collectively, our results demonstrate the importance of Casparian strips in preventing apoplastic diffusion of boric acid into the stele under excess supply.

## Introduction

Boron (B) is an essential micronutrient for plants, with a narrow concentration range for optimal growth. B deficiency causes various defects, such as decreased root elongation and fertility (Dell and Huang, 1997). These effects are mainly dependent on the function of B in crosslinking pectin at rhamnogalacturonan II regions (Kobayashi et al., 1996). B toxicity also causes tissue necrosis, chlorosis, and reduced growth (Nable et al., 1997). Excess B affects various cellular metabolism processes and leads to accumulation of reactive oxygen species (ROS) and DNA damage (Landi et al., 2019). Many studies have reported that necrosis first occurs at the leaf tip and margin due to B accumulation at the end of the transpiration stream (Reid et al., 2004). The very narrow concentration tolerance range is attributed to the chemical form of B in solution. Boric acid, the major chemical form of B in solution, is a small, neutral molecule with relatively high membrane permeability. When its concentration is sufficient in soil solution, it can be taken up by root cells and translocated into the xylem vessels through passive diffusion. However, plants possess mechanisms to control B transport when its availability is limited or excessive in soils (Yoshinari and Takano, 2017).

When water and nutrients are taken up by plant roots, they are transported through several cell layers, including the epidermis, cortex, and endodermis, and then to the xylem vessels for translocation to aboveground tissues. Radial transport in roots consists of three pathways: the apoplastic (diffusion through the apoplastic space), symplastic (diffusion through plasmodesmatal connections), and coupled transcellular pathways (vectorial transport through a polarized importer and exporter) (Barberon, 2017). Under B limitation, efficient radial transport of B is largely dependent on a coupled transcellular pathway consisting of the polarly localized boric acid channel NIP5;1 and the borate exporters BOR1 and BOR2 (Takano et al., 2002; Takano et al., 2006; Miwa et al., 2013; Yoshinari and Takano, 2017). When the soil contains high levels of B, these transporters are downregulated to avoid uptake and translocation of excess B. The downregulation of NIP5;1 depends on B-induced ribosome stalling at AUG-stop in the 5′-untranslated region (UTR) of *NIP5;1* mRNA, resulting in translational inhibition and mRNA degradation (Tanaka et al., 2011; Tanaka et al., 2016). The translation of BOR1 is also repressed under high B concentrations, dependent on the 5′-UTR (Aibara et al., 2018). In addition, high B supply induces rapid internalization and vacuolar degradation of BOR1 (Takano et al., 2005) and BOR2 (Miwa et al., 2013). In plants including barley and rapeseed, a linear relationship between B concentrations in medium and in plant tissues has been reported under sufficient to excess B supply (Marschner, 1995). This phenomenon can be interpreted as a combination of passive apoplastic transport and passive membrane transport of boric acid through diffusion and nonspecific channels. In *Arabidopsis*, another borate exporter, BOR4, is upregulated in response to high B supply and is expressed at least in epidermal cells in the root tip region and endodermis in mature root regions (Miwa et al., 2014). BOR4-GFP is localized to the outer plasma membrane of root epidermal cells (Miwa et al., 2007). BOR4 overexpression decreases B accumulation in roots and shoots, and increases tolerance to excess B (Miwa et al., 2007). Conversely, *bor4* knockout increases B accumulation in shoots and decreases tolerance to excess B (Miwa et al., 2014). Therefore, excessive transport of B from the root surface to the stele can be at least partly prevented by BOR4-dependent borate efflux.

In the roots of higher plants, Casparian strips, which are ring-like structures consisting of lignin, seal the apoplastic spaces between endodermal cells. They have long been thought to function as a barrier blocking the apoplastic diffusion of water and solutes. However, recently, it became possible to test this hypothesis in *Arabidopsis* mutants (Hosmani et al., 2013; Pfister et al., 2014; Kamiya et al., 2015; Doblas et al., 2017; Li et al., 2017; Nakayama et al., 2017; Duan et al., 2018; Wang et al., 2019; Calvo-Polanco et al., 2021; Reyt et al., 2021). For example, plants with mutations of *ENHANCED SUBERIN 1* (*esb1*) and *CASPARIAN STRIP MEMBRANE DOMAIN PROTEINs* (*casp1 casp3*) showed altered shoot ionomes containing more sulfur, potassium (K), and molybdenum (Mo), and less magnesium (Mg), calcium (Ca), manganese (Mn), and iron (Fe) than the wild-type (WT), but normal levels of B (Hosmani et al., 2013). Similarly, the leaves of a mutant of *myb36*, a transcription factor regulating the expression of *CASP1*, *ESB1*, and *Peroxidase 64* (*PER64*), accumulated more sodium (Na), Mg, and zinc (Zn), and less Ca, Mn, Fe, and B, than Col-0 (Kamiya et al., 2015). Mutation of another Casparian strip-related gene, *LORD OF THE RINGS 1* (*LOTR1*), also affected the concentrations of numerous elements in leaves, including increased B levels (Li et al., 2017). However, these mutants exhibited enhancement of suberization, another diffusion barrier around mature endodermal cells.

In this study, we examined the role of Casparian strips in B transport, particularly under excess B conditions, using the mutants *sgn3* and *sgn4*. The receptor-like kinase SCHENGEN3 (SGN3), also known as GASSHO1 (GSO1), is required to localize CASPs into a ring-like domain (Pfister et al., 2014). Among mutants with defective Casparian strip formation, *sgn3* mutants are suitable for analyzing Casparian strip function because they have discontinuous strips with unaffected suberin deposition (Pfister et al., 2014). NADPH oxidase SGN4/respiratory burst oxidase homolog F (RBOHF) is required for the localized deposition of lignin in the Casparian strip domain. In loss-of-function mutants of *SGN4*, Casparian strips were absent in younger root parts, whereas aberrantly structured strips and slightly elevated suberin deposition levels were observed in older root parts (Lee et al., 2013; Fujita et al., 2020). Surprisingly, a previous study showed that *sgn3* mutants are able to maintain growth and WT levels of various nutrient elements including Ca, B, Fe, Mn, copper (Cu), Mo, cobalt, and Na under standard laboratory conditions (Pfister et al., 2014). However, they consistently show reduced K and increased Mg and cesium (Cs) levels, and tend to have reduced Zn levels in shoots under various conditions. They also show enhanced sensitivity to K deficiency. Although the B concentration in shoots of the *sgn3* mutants was not largely affected under normal growth conditions (Pfister et al., 2014), the Casparian strip mutants may be unable to maintain B homeostasis when excess boric acid is supplied. Our study using the *sgn3* and *sgn4* mutants demonstrates that the Casparian strip functions to restrict the flow of excess boric acid into the root stele.

## Materials and methods

### Plant materials and growth medium

We obtained the *Arabidopsis* T-DNA insertion mutants *sgn3–3* (SALK_043282), *sgn3–4* (SALK_064029), and *rbohf-3* (SALK_059888) from the *Arabidopsis* Biological Resource Center. The *sgn4–1* mutant was isolated from an ethyl-methane sulfonate (EMS)-treated population in a previous study (Lee et al., 2013). The composition of the plant growth medium is as follows: 1.77 mM sodium phosphate buffer (pH 5.8), 1.5 mM MgSO_4_, 2.0 mM Ca(NO_3_)_2_, 3.0 mM KNO_3_, 50 μM Fe-EDTA, 10.3 μM MnSO_4_, 1.0 μM ZnSO_4_, 1.0 μM CuSO_4_, 130 nM CoCl2, and 24 nM (NH_4_)_6_Mo_7_O_24_ (Takano et al., 2005; Yoshinari and Takano, 2020). The control level of B is 30 µM boric acid. In each experiment, various concentrations of boric acid were supplied.

### Growth analysis

For growth analysis in plates, seeds were surface-sterilized and sown on the medium containing 1% sucrose and solidified with 1% gellan gum (FUJIFILM Wako Chemicals) in a sterile square Petri dish (140 × 100 mm; Eiken, Tokyo). Boric acid was supplemented at the indicated concentrations (0.1, 1, 30, 3,000 or 6,000 μM). After 2–3 days of 4°C treatment in the dark, the plates were placed vertically in a growth chamber at 22°C with a 16 h light/8 h dark cycle under fluorescent lamps with a light intensity of 110 μmol m^−2^ s^−1^. Primary root length and shoot area of the 10-day-old plants were measured using the ImageJ software (https://imagej.nih.gov/ij/). For each data point, 11–16 plants were analyzed.

For growth analysis in hydroponic culture (Takano et al., 2001), plants were grown for 5 weeks with 1.4 L liquid medium containing boric acid at the indicated concentrations (0.1, 1, 10, 30, 100, 300 or 1,000 μM). The environmental parameters of the growth chamber were as follows: 9 h/15 h light/dark cycle, 22°C, fluorescent lamps with a light intensity of 110 μmol m^−2^ s^−1^, and 65–75% humidity. For each B concentration and genotype, 4–5 plants were analyzed.

For growth analysis on rockwool, seeds were surface-sterilized and soaked in sterile water. After 2–3 days of 4°C treatment in the dark, three seeds were sown at each of the four corners of a rockwool block (36 × 36 × 40 mm, Grodan, The Netherlands), placed in containers (35 cm × 12 cm), and supplied with Milli-Q water. The environmental parameters of the growth chamber were as follows: 9 h/15 h light/dark cycle, 22°C under fluorescent lamps with a light intensity of 110 μmol m^−2^ s^−1^, and 35–70% humidity. The seedlings were grown for 1 week and then two of the three plants were removed from each corner, leaving the healthiest one. Next, the plants were grown with liquid medium containing boric acid at the indicated concentrations (10, 30, 100, 300, 1,000 or 2,000 μM). After 3 weeks, the two smaller plants in each rockwool block were removed. After another week, the shoots of the 5-week-old plants were harvested and weighed. For each B concentration and genotype, 5–6 plants were analyzed.

### Measurement of B accumulation in shoots

Plants were grown in hydroponic culture as described above. The shoots were collected and dried in a forced-air dryer (WFO-700; Eyela, Japan). Dry weights were measured and the samples were digested in concentrated nitric acid for boron determination (FUJIFILM Wako Chemicals). B quantification was conducted based on the curcumin assay (Mohan and Jones, 2018). For each B concentration and genotype, 4–5 plants were analyzed.

### Tracer analysis

Col-0 and the Casparian strip mutants were grown hydroponically as described previously (Takano et al., 2001). The environmental parameters of the growth chamber were as follows: 9 h/15 h light/dark cycle, and 22°C under fluorescent lamps with a light intensity of 110 μmol m^−2^ s^−1^. The plants were supplied with MilliQ water for 1 week and then with liquid medium containing 1 µM ^11^B-enriched boric acid (99%, Cambridge Isotope Laboratories) for 3 weeks. The plants were incubated with liquid medium containing 300 µM ^10^B-enriched boric acid (96%, Cambridge Isotope Laboratories) for 1, 2, and 4 h and the shoots and roots were collected. As a control, shoots and roots were collected without incubation with ^10^B-enriched boric acid. The shoots were dried in a forced-air dryer at 70°C for longer than 2 days and then weighed. The samples were digested with 3 mL concentrated nitric acid for boron determination (Wako Pure Chemical) in 50 mL DigiTUBEs (GL Sciences, Tokyo, Japan) at 110°C using a DigiPREP MS apparatus (GL Sciences). After the nitric acid solution evaporated, 20 mL 2% nitric acid containing 5 ppb beryllium as internal standard was added to dissolve the samples. ^10^B concentrations were measured via inductively coupled plasma mass spectrometry (ICP–MS, ICPM-8500, SHIMADZU, Kyoto, Japan). Shoots or roots from 4 plants were treated together as one sample. For each genotype and time point, 4 samples were analyzed.

### Ionome analysis

Plants were grown hydroponically for 25 days as described previously (Pfister et al., 2014), with liquid medium containing boric acid at concentrations of 0.3, 30, and 1000 µM. After 15 days of growth, the plants were additionally supplied with 3 µM CsCl and 10 µM SrCl_2_. The environmental parameters of the growth chamber were as follows: 10 h/14 h light/dark cycle, 22°C under fluorescent lamps, and 70% humidity. Both experiments included results already described in a previous report (for 30 µM Col-0, *sgn3–3*, and *sgn3–4*). Sample preparation and analysis by ICP–MS (ELAN, DRC-e; Perkin–Elmer, Waltham, MA, USA) were as described previously (Pfister et al., 2014).

### Plasmid construction

The *proUBQ10:NIP5;1 5*′*UTR : ELuc-PEST* plasmid (pSW52) was previously reported (Fukuda et al., 2018). For construction of the *proCIF2:NIP5;1 5*′*UTR : ELuc-PEST* plasmid (pJK8), we amplified the 1,756 bp upstream sequence of the CIF2 coding region from the Col-0 genome using the following primers: 5′-GCTCAAGCTAAGCTTATTGTGAAAGTCACACACTCG-3′ and 5′-TGAGCTTATGTCTAGCTTCTTTCTCCTTTCAATTTTTGATG-3′. *NIP5;1 5*′*UTR : ELuc-PEST* sequence was amplified from pSW52 using the following primers: 5′-CTAGACATAAGCTCAAAGACTAACCAAACC-3′ and 5′-CCCTGCAGGGGATCCCTCAAGAATGGCATCTACACATTGATC-3′. These two fragments were subcloned into a pPZP-based binary vector (Hajdukiewicz et al., 1994) containing the HSP terminator sequence (a gift from Yuji Iwata, Osaka Metropolitan University) using the BamHI and HindIII restriction enzymes and the In-Fusion HD cloning kit (Takara, Kusatsu, Japan).

### Generation of transgenic plants

Plasmid binary vectors were introduced into *Arabidopsis* Col-0 plants using the floral dip method (Clough and Bent, 1998) with *Agrobacterium* strain GV3101:pMP90. To generate the Casparian strip mutants expressing the stele-specific biosensor for boric acid, *sgn3–3* and *sgn4–1* were crossed with *proCIF2:NIP5;1 5*′*UTR : ELuc-PEST/*Col-0 or *proUBQ10:NIP5;1 5*′*UTR : ELuc-PEST/*Col-0 plants. F2 plants were genotyped by polymerase chain reaction (PCR), and F3 seeds from F2 homozygous mutant plants were used for chemiluminescence imaging experiments.

### Luciferase chemiluminescence imaging

Transgenic plants expressing boric acid biosensors were grown on plates of solid medium containing 0.5 μM B, which were placed vertically in a growth chamber at 22°C under fluorescent lamps with a 16 h/8 h light/dark cycle and light intensity of 90 μmol m^−2^ s^−1^. To visualize the tissue-specific expressing pattern of the biosensors, 5–6-day-old plants were placed between two cover glasses (24 × 40 and 25 × 60 mm, Matsunami, Kishiwada, Japan) with liquid medium containing 0.5 μM B and 100 μM D-luciferin (FUJIFILM, Wako Chemicals) and observed under an inverted microscope (Axiovert.A1, Carl Zeiss, Oberkochen, Germany) equipped with an electron-multiplying charge-coupled device (EMCCD) camera (Evolve, Photometrics, Tucson, AZ, USA) using a 5× objective lens. For time-course analysis under the inverted microscope equipped with the EMCCD camera, 6- to 10-day-old plants with 1.5–2.0 cm root were incubated in liquid medium containing 0.5 μM B and 100 μM D-luciferin for 1 h. These plants were transferred onto glass bottom dishes (27 mm diameter, AGC Techno Glass, Haibara, Japan) and the roots were covered by solidified (0.8% gellan gum) medium containing 300 μM B and 100 μM D-luciferin and liquid medium containing 300 μM B and 100 μM D-luciferin. Images of the region 5 mm from root tips were taken using a 5× objective lens every 10 min and the exposure time was set to 10 min. For each genotype, four plants were analyzed.

For time-course analysis using a chemiluminescence imaging system, 6- to 7-day-old plants were incubated in liquid medium containing 0.5 μM B and 100 μM D-luciferin for 1 h. These plants were transferred onto solidified (0.3% gellan gum) medium containing 300 μM B and 100 μM D-luciferin and visualized using the FUSION chemiluminescence imaging system (Vilber, Paris, France). Images were taken every 5 min and the exposure time was set to 1 min (0–5 mm from root tips) or 5 min (5–10 mm from root tips). The signals from five plants were analyzed for each genotype.

### Statistical analysis

Statistical analyses were performed using GraphPad Prism 8. P values were determined using the Student’s *t*-test or one-way ANOVA with Dunnett’s *post-hoc* test.

## Results

### *sgn3* and *sgn4* mutants show reduced tolerance to high B concentrations

**Figure 1:**
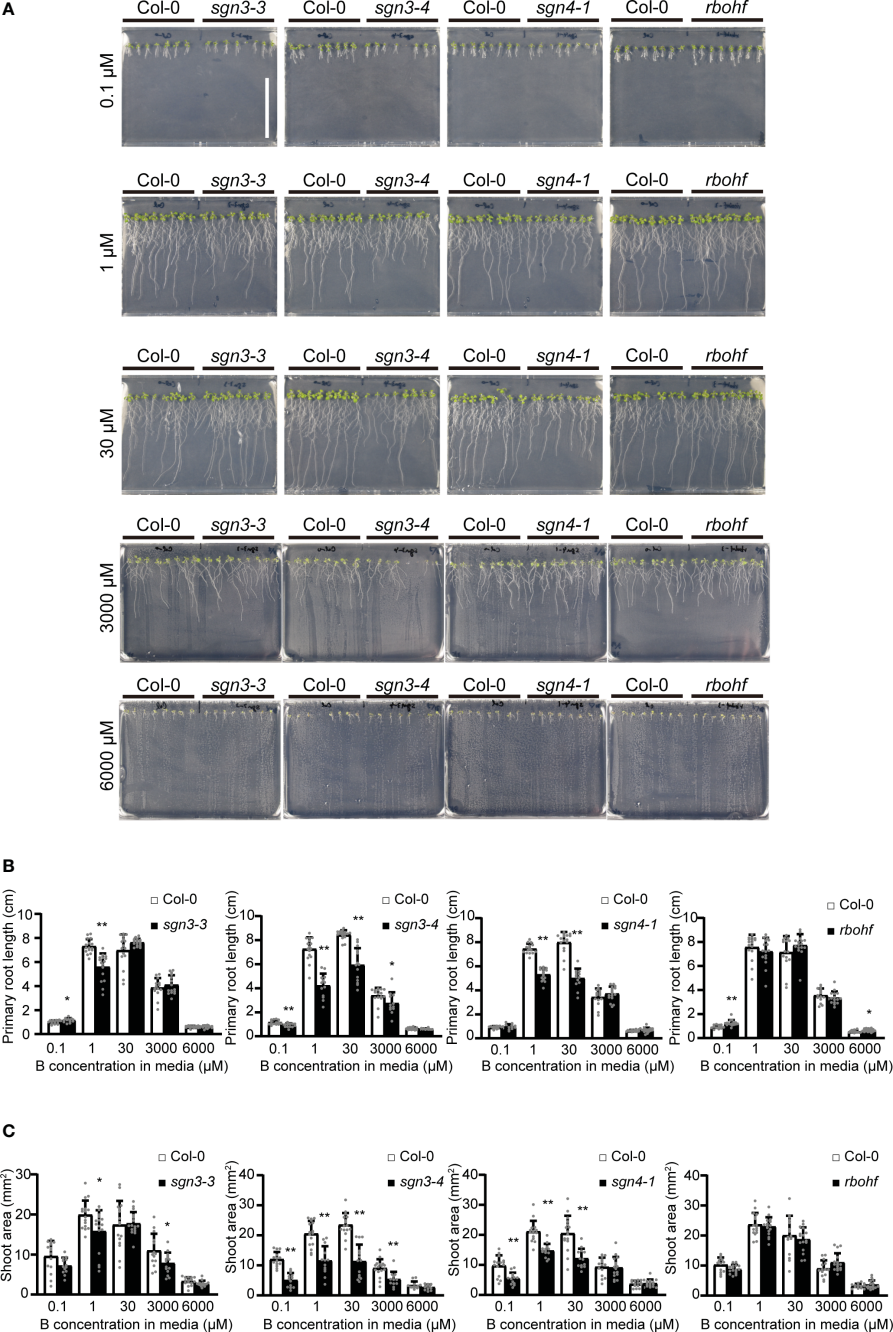
Growth of Casparian strip mutants under various boron **(B)** concentrations in plate culture. **(A)** Representative photographs of Col-0, *sgn3–3*, *sgn3–4*, *sgn4–1*, and *rbohf* plants grown for 10 days on medium containing 0.1, 1, 30, 3000, or 6000 μM of boric acid. Scale bar = 5 cm. **(B)** Root lengths of plants grown as described in **(A)**. Data are means ± standard deviations (SDs) (n = 11–16). Asterisks indicate significant differences between Casparian strip mutants and Col-0 (Student’s *t*-test, *P < 0.05, **P < 0.01). **(C)** Shoot areas of plants grown as described in **(A)**. Data are means ± SDs (n = 11–16). Asterisks indicate significant differences between Casparian strip mutants and Col-0 (Student’s *t*-test, *P < 0.05, **P < 0.01).

To elucidate the roles of Casparian strips in plant growth under low- and high-B conditions, we investigated the growth of *Arabidopsis* mutants with defective strips. Col-0, *sgn3–3*, *sgn3–4*, *sgn4–1*, and *rbohf* (a *sgn4* mutant) plants were grown on plates of solid media containing various concentrations (0.1 μM, 1 μM, 30 μM, 3 mM, or 6 mM) of boric acid. As reported previously, these plants did not show severe growth defects under standard laboratory growth conditions (Pogány et al., 2009; Pfister et al., 2014) (**Figure 1**). The mutant plants showed variable growth of roots (**Figures 1A, B**) and shoots (**Figures 1A, C**) under low-B conditions (0.1 and 1 µM) and high-B conditions (3 and 6 mM) compared to the wild-type Col-0, while there was no clear tendency.

**Figure 2:**
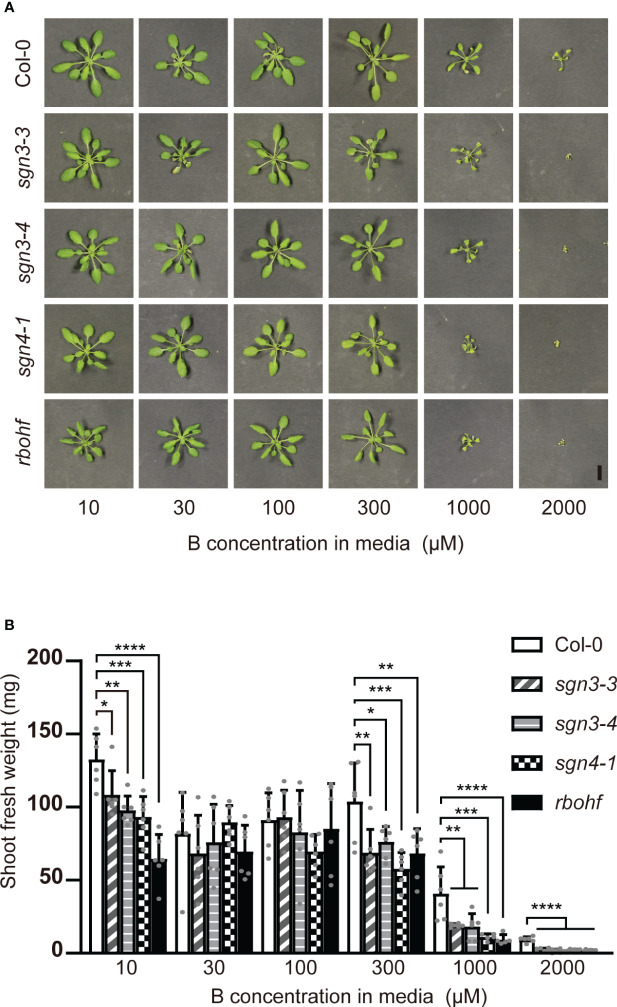
Growth of Casparian strip mutants under various B concentrations on rockwools. **(A)** Representative photograph of Col-0, *sgn3–3*, *sgn3–4*, *sgn4–1* and *rbohf* plants grown for 5 weeks on rockwool with liquid medium containing 10, 30, 100, 300, 1000, or 2000 μM boric acid. Scale bar = 1 cm. **(B)** Fresh weights of shoots from plants grown as described in **(A)**. Data are means ± SDs (n = 5–6). Asterisks indicate significant differences between mutant plants and Col-0 (one-way ANOVA with Dunnett’s *post-hoc* test, *P < 0.05, **P < 0.01, ***P < 0.001, ****P < 0.0001).

As B is mainly transported via the transpiration stream from roots to shoots, B toxicity should be apparent under high-transpiration conditions. The high humidity of plates inhibits the transpiration stream, and we therefore examined the effect of B concentration on the growth of mutant plants on rockwool blocks placed in containers and supplied with liquid medium containing various concentrations (10, 30, 100, 300, 1,000, or 2,000 μM) of boric acid for 5 weeks (**Figure 2**). With 10 µM B supply, the fresh weights of shoots of the *sgn3–3, sgn3–4, sgn4–1* and *rbohf* mutants were reduced compared to Col-0 (82%, 74%, 70% and 49%). Under normal to moderately high B conditions (30 and 100 μM), the growth of mutants was comparable to that of Col-0 except for *sgn4–1* at 100 μM B (76% of Col-0 growth). However, the growth of these mutants consistently showed significant reductions under moderately high to excess B (300 μM, 1 mM or 2 mM) conditions (66%, 73%, 56% and 66% at 300 μM B; 47%, 45%, 27% and 23% at 1 mM B; and 26%, 17%, 24% and 19% at 2 mM B, respectively).

We also grew the mutant and wild-type plants in a hydroponic system with liquid medium containing various concentrations (0.1, 1, 10, 30, 100, 300 or 1,000 μM) of boric acid for 5 weeks (**Supplementary Figures 1A, B**). Although the rates of growth reduction were different in the different experiments, the sensitivity of Casparian strip mutants to excess B under high-transpiration conditions was confirmed.

Collectively, the mutants of *SGN3* and *SGN4/RBOHF* exhibited reduced growth under various B conditions, and this trend was particularly evident with excess B.

### *sgn3* and *sgn4* mutants translocate more B toward shoots under excess B conditions

We previously reported the shoot ionomes (elemental profile) of *sgn3* mutants grown in hydroponic culture with sufficient B supply (30 µM). In this study, we compared the shoot ionomes of *sgn3–3*, *sgn3–4*, and *rbohf* mutants under limited (0.3 µM), sufficient (30 µM), and excess (1 mM) B conditions in two independent experiments (**Supplementary Figure 2**; data for three B conditions were collected in the same experiments; results for 30 µM B were reported by Pfister et al., 2014). Regardless of B supply, these mutants accumulated significantly more Mg than the Col-0 WT in both experiments and significantly more Zn and less K in the first experiment but not in the second experiment. Cs concentrations were consistently higher in Casparian strip mutants, although the difference was not statistically significant in the second experiment. Notably, B limitation and excess did not consistently alter the ionome pattern, except for B in the WT and Casparian strip mutants in both experiments. Under sufficient B (30 μM) supply, B accumulation in the shoots of *sgn3–3*, *sgn3–4*, and *rbohf* mutants showed no significant difference from that in Col-0. Under limited B supply (0.3 µM), such shoots contained comparable or slightly lower concentrations of B than Col-0. Under high B (1 mM) conditions, *sgn3–3*, *sgn3–4*, and *rbohf* mutants tended to show higher B accumulation (86%, 53%, and 91% more than Col-0 in the first experiment and 71%, 35%, and 29% more than Col-0 in the second experiment, respectively), although the difference was statistically significant only in *sgn3–3* and *rbohf* in the first experiment. These results suggest that B homeostasis cannot be maintained under excess B conditions in Casparian strip mutants.

**Figure 3:**
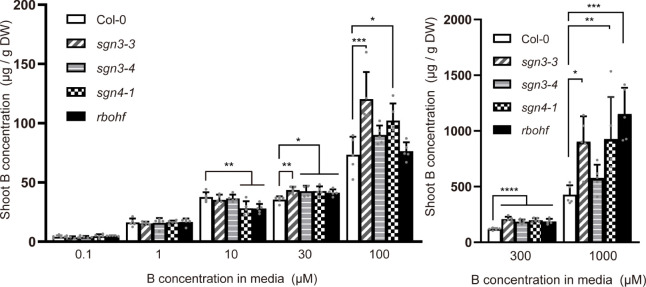
Accumulation of B in Casparian strip mutants grown in hydroponic culture. B concentrations in shoots of Col-0, *sgn3–3*, *sgn3–4*, *sgn4–1*, and *rbohf* plants grown hydroponically for 5 weeks with liquid medium containing 0.1, 1, 10, 30, 100, 300, or 1,000 μM boric acid. Data are means ± SDs (n = 4–5) Asterisks indicate significant differences between mutant plants and Col-0 (one-way ANOVA with Dunnett’s *post-hoc* test, *P < 0.05, **P < 0.01, ***P < 0.001, ****P < 0.0001).

To further investigate B transport in *sgn3* and *sgn4* mutant plants under low-to-high B conditions, we quantified B concentrations in the shoots of plants grown hydroponically with 0.1, 1, 10, 30, 100, 300, and 1,000 µM B for 5 weeks (**Supplementary Figure 1**). At low B supply levels (0.1 and 1 µM B), the B concentrations in the shoots of *sgn3* and *sgn4* mutants were comparable to those of Col-0 plants (**Figure 3**). However, at normal and moderately higher B supply levels (above 30 µM), *sgn3–3, sgn3–4, sgn4–1* and *rbohf* mutants showed higher B accumulation than Col-0 although some values were not statistically significant. These results indicate that the Casparian strip plays a role in restricting the accumulation of B in shoots under high-B conditions.

**Figure 4:**
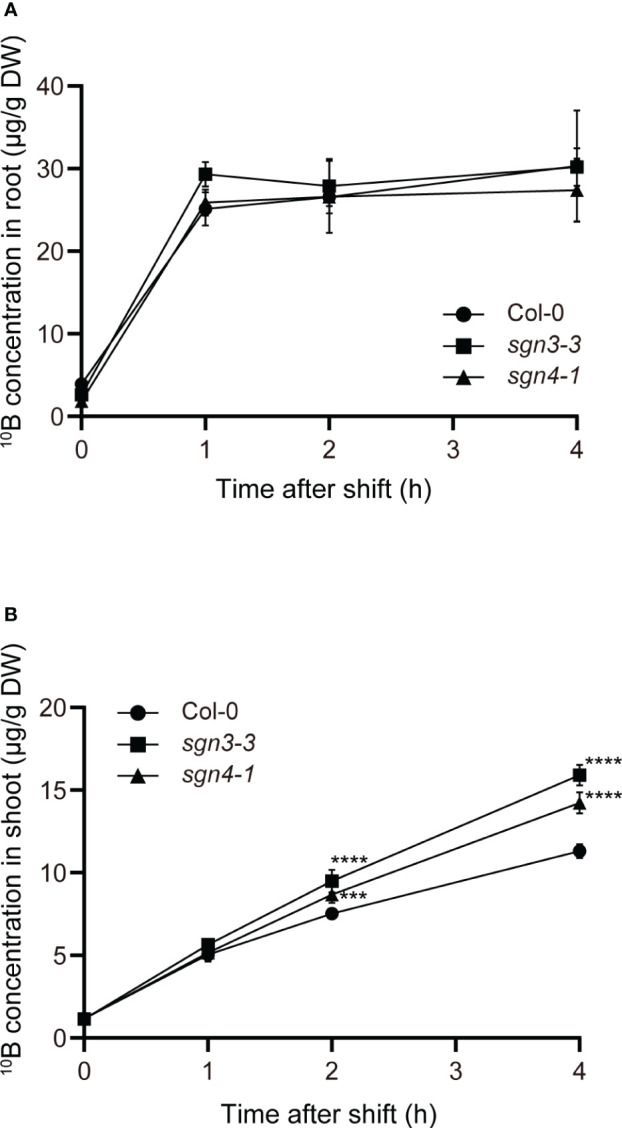
Time-course analysis of uptake and translocation of B in Casparian strip mutants grown in hydroponic culture. **(A, B)** Time-couse of B uptake and translocation using stable B isotopes. The ^10^B concentrations in Col-0, *sgn3–3*, or *sgn4–1* roots **(A)** and shoots **(B)** after 0, 1, 2, and 4 h incubation with 300 μM ^10^B-enriched boric acid are shown. Data are means ± SDs (n = 4). Asterisks indicate significant differences between mutant plants and Col-0 (two-way ANOVA with Dunnett’s *post-hoc* test, ***P < 0.001, ****P < 0.0001).

Next, we conducted a time-course tracer analysis using stable B isotopes to examine the role of Casparian strips in the uptake and translocation of B under a sufficient or slightly excess B supply (**Figure 4**). The Col-0 WT and the Casparian strip mutants were grown hydroponically for 4 weeks with 1 µM ^11^B-enriched boric acid (99%). The growth of the plants was normal under this condition as shown in **Supplementary Figure 1** (1 µM). The plants were transferred to medium containing 300 μM ^10^B-enriched boric acid (96%), and incubated for 1, 2, and 4 h. Then, roots and shoots were sampled and digested, and ^10^B concentrations were measured via ICP–MS. For the control (time 0), plants were sampled without being transferred to medium containing ^10^B-enriched boric acid. Both in Col-0 and mutant plants, root ^10^B concentrations were saturated after the increase within the first 1 h (**Figure 4A**). There was no significant difference in root ^10^B concentrations between the mutants and Col-0. On the other hand, shoot ^10^B concentrations increased over time and *sgn3–3* and *sgn4–1* mutant plants accumulated significantly higher ^10^B concentrations than Col-0 at 2 and 4 h (**Figure 4B**). The tracer concentrations in *sgn3–3* and *sgn4–1*, calculated by subtracting ^10^B concentrations in the control (time 0) from those in ^10^B-treated plants, were 31% and 18% higher than those in Col-0 at 2 h and 46% and 29% higher at 4 h, respectively. These results demonstrate that the *sgn3* and *sgn4* mutants translocate larger amounts of B to their shoots than Col-0 plants. Collectively, these results suggest that the Casparian strip is functional in restraining the translocation of boric acid under high-B conditions.

### Boric acid is rapidly diffused into the stele in *sgn3* and *sgn4* mutants

The Casparian strip is a structure that forms between root endodermal cells. Therefore, higher B translocation into shoots in the *sgn3* and *sgn4* mutants was thought to be a consequence of increased apoplastic diffusion of boric acid into the stele. To visualize this process, we utilized a genetically encoded biosensor for cytosolic boric acid (Fukuda et al., 2018). This biosensor encodes *NIP5;1* 5′UTR and the luciferase gene Emerald Luc (*ELuc*), which is fused with the protein degradation signal PEST under the control of the *UBQ10* promoter. When B levels in the cytosol are high, the expression of *ELuc* is reduced by *NIP5;1* 5′UTR-dependent translational repression and mRNA degradation (Tanaka et al., 2011; Tanaka et al., 2016). In tobacco BY-2 cells, this *NIP5;1* 5′UTR-based biosensor with a fluorescence protein reporter responded to intracellular B in a range of 30–500 µM (Fukuda et al., 2018). Notably, this lower limit is overestimated due to the effect of B deficiency in tobacco BY-2 cells. In a wheat germ extract *in vitro* translation system, *NIP5;1 5*′*UTR : LUC* responded to >10 µM B (Tanaka et al., 2016). We introduced the *proUBQ10:NIP5;1 5*′*UTR : Eluc-PEST* construct into the *sgn3–3* mutants by crossing with transgenic Col-0 plants and performed a time-course analysis of chemiluminescence by transferring the plants from medium containing 0.5 µM B and D-luciferin to that containing 300 μM B and D-luciferin (**Supplementary Figure 3**). In the mature root region where Casparian strips are established in the wild-type plants (5 mm from root tips), chemiluminescence signals similarly decreased in the transgenic Col-0 and *sgn3-3* plants (74% and 81% within 90 min), indicating the similar increase of boric acid concentrations in the majority of root cells (**Supplementary Figure 3**). Quantification of the signals in outer cell layers (epidermal and cortical cells) in this region also showed similar signal decrease between the genotypes (75% and 79% within 90 min, **Supplementary Figure 3**). These results support that the defect in Casparian strips does not affect changes of boric acid concentrations in outer cell layers of the roots upon high-B supply.

**Figure 5:**
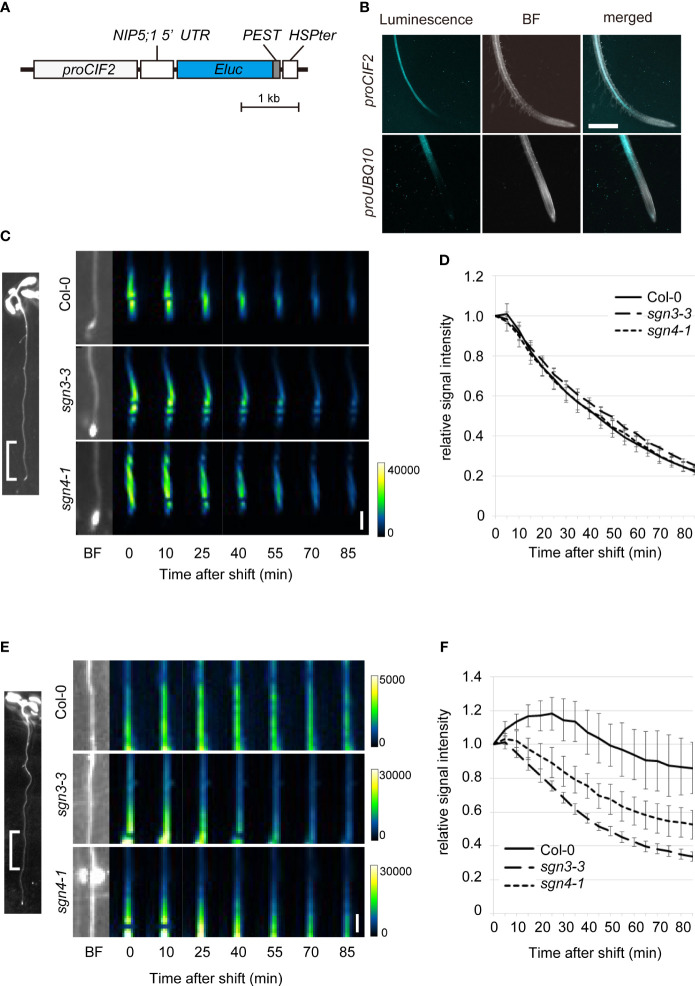
Time-course analysis of boric acid flux into the root stele of Casparian strip mutants using a biosensor genetically encoded for boric acid. **(A)** Schematic diagram of the construct of a stele-specific biosensor for boric acid. The mRNA level of *NIP5;1 5*′*UTR ELuc-PEST* is decreased with higher cytoplasmic boric acid concentration. The PEST protein degradation signal renders short life-time of the reporter protein. *CIF2* promoter drives expression of the RNA-based biosensor in the root stele. **(B)** Chemiluminescence in the transgenic Col-0 root expressing the boric acid biosensor expressed under the control of the *CIF2* promoter (*proCIF2:NIP5;1 5*′*UTR : ELuc-PEST*, upper) or *UBQ10* promoter (*proUBQ10:NIP5;1 5*′*UTR : ELuc-PEST*, lower). Scale bar = 1 mm. BF, bright field. **(C)** Representative images of luminescence at 0–5 mm from the root tips of transgenic Col-0, *sgn3–3*, or *sgn4–1* plants expressing the boric acid biosensor under the control of the stele-specific *CIF2* promoter after transfer to media containing 300 μM boric acid. Representative BF image showing the analyzed root region is displayed on left. Images were taken by a chemiluminescence imaging system. Scale bar = 1 mm. **(D)** Time course of changes in relative signal intensity at 0–5 mm from the root tips of transgenic Col-0, *sgn3–3*, or *sgn4–1* plants after transfer to medium containing 300 μM boric acid. Relative signal intensity was calculated by dividing each signal by the initial intensity for each plant. Data are means ± SDs (n = 5). The relative signal intensities in *sgn3–3* and *sgn4–1* were not significantly different from those in Col-0 except for that in *sgn3-3* at 50 min (*P* < 0.05 by one-way ANOVA with Dunnett’s *post-hoc* test). **(E)** Representative images of luminescence at 5–10 mm from the root tips of transgenic Col-0, *sgn3–3*, or *sgn4–1* plants after transfer to medium containing 300 μM boric acid. A representative image of the analyzed root region is displayed on the left. Images were taken by a chemiluminescence imaging system. Scale bar = 1 mm. **(F)** Time course of changes in relative signal intensity at 5–10 mm from the root tips of transgenic Col-0, *sgn3–3*, or *sgn4–1* plants after transfer to medium containing 300 μM boric acid. Relative signal intensity was calculated by dividing each signal by the initial intensity for each plant. Data are means ± SDs (n = 5). “Time after shift” represents the time since exposure began (1 min for C and D, 5 min for E and F). The relative signal intensities in *sgn3–3* and *sgn4–1* were consistently lower than those in Col-0 at 20 min and later (*P* < 0.0001 by one-way ANOVA with Dunnett’s *post-hoc* test).

To visualize changes related to boric acid concentration specifically in the stele, we utilized the promoter of the *CASPARIAN STRIP INTEGRITY FACTOR* (*CIF2*) gene to express the biosensor construct in stele cells in the root elongation and differentiation zones (**Figure 5A**) (Doblas et al., 2017; Nakayama et al., 2017). We introduced this biosensor (*proCIF2:NIP5;1 5*′*UTR : Eluc-PEST*) to the Col-0 plants and observed chemiluminescence on solid medium containing a low concentration of B (0.5 µM) and D-luciferin. Whereas the *UBQ10* promoter-driven biosensor showed signals in whole cell layers of the root, the *CIF2* promoter-driven biosensor showed signals exclusively in the stele (**Figure 5B**).

To examine the role of Casparian strips in the apoplastic diffusion of boric acid into the stele, we introduced the *proCIF2:NIP5;1 5*′*UTR : Eluc-PEST* construct into the *sgn3–3* and *sgn4–1* mutants by crossing with transgenic Col-0 plants. When the biosensor-expressing plants were transferred from medium containing 0.5 µM B to that containing 300 μM B, strong signals observed in the tip-adjacent regions (0–5 mm from root tips) decreased sharply over time (80% within 90 min) in both the Col-0 and mutant backgrounds (**Figures 5C, D**). This indicates rapid translocation of B into the stele of root tips, without the development of Casparian strips. In mature regions (5–10 mm) in Col-0 background plants, the signals increased and then decreased gradually (approximately 14% within 90 min), reflecting a slow increase in the B concentration in stele cells (**Figures 5E, F**). Importantly, luminescence decreased faster in the *sgn3–3* and *sgn4–1* mutant plants than in Col-0 (66% and 47%, respectively, within 90 min), indicating the faster increase of the B concentration in the stele cells of the mutants. These results strongly support the hypothesis that the Casparian strip plays a role in preventing apoplastic diffusion of excess boric acid into the stele.

## Discussion

Previous studies of Casparian strip mutants have revealed altered ionomic profiles, suggesting that the apoplastic barrier is important for maintaining elemental homeostasis in plants (Hosmani et al., 2013; Kamiya et al., 2015; Li et al., 2017). However, many of the mutants used in these studies exhibited altered suberization, which may mask the role of the Casparian strips. *sgn3* mutants are ideal for this purpose because they show no significant alterations other than discontinuous Casparian strips (Pfister et al., 2014). Using these mutants and *sgn4* mutants, in which the formation of Casparian strips is delayed and the suberin deposition is slightly enhanced (Lee et al., 2013; Fujita et al., 2020), we examined the role of Casparian strips under various B conditions. *sgn3* and *sgn4* mutant plants showed reduced shoot growth, particularly under excess B conditions in hydroponic and rockwool culture (**Figure 2**, **Supplementary Figure 1**). These mutants showed increased B accumulation in shoots and faster translocation of B into the root stele and shoots when excess B was supplied (**Figures 3** **–** **5**, **Supplementary Figures 2**). These results reveal the role of Casparian strips in excess B tolerance, exerted through limitation of apoplastic diffusion of B into the stele. Other recent studies have demonstrated roles of Casparian strips in tolerance of stressful conditions. Maize and *Arabidopsis* mutant plants of the *ESB-Like* (*ESBL*) gene showed greater accumulation of Na in shoots and sensitivity to NaCl stress (Wang et al., 2022). Earlier formation of Casparian strips induced by the overexpression of *OsCASP1* in rice led to reduced Ca accumulation in shoots and improved tolerance to high concentrations of Ca (Wang et al., 2021). *SbCASP4* overexpression in *Arabidopsis* enhanced Casparian strip formation, improved tolerance to salinity stress and reduced the Na concentration in shoots (Wei et al., 2021). An *Arabidopsis cif1 cif2* double mutant with discontinuous Casparian strips was reportedly sensitive to excess iron and accumulated high levels of iron in its xylem sap (Nakayama et al., 2017). Collectively, these studies and our results show that a functional apoplastic barrier is essential for tolerance of the harmful levels of elements such as B, Ca, Na, and Fe. It should be noted that our ionomic analysis showed altered accumulation of ions such as increased Mg, Cs and reduced K, Zn in *sgn3* and *sgn4* mutants (Pfister et al., 2014, **Supplementary Figure 2**). It will be interesting to test the roles of Casparian stirps in tolerance of harmful levels of various elements.

In the present study, the *sgn3* and *sgn4* mutants showed no clear alteration of growth compared to WT plants when cultured on plates containing as much as 3 mM B, although their growth was reduced in the hydroponic culture containing 1 mM B. The main reason for this difference is likely the effect of transpiration. In a recent report, *sgn3–3* and *sgn3–3 myb36-2* mutants showed normal root hydraulic conductivity and altered ionomic profiles (Reyt et al., 2021). These mutants showed elevated death rates when grown at 60% relative humidity, whereas almost all plants survived at 80% relative humidity. Ionomic responses to low humidity observed in Col-0 leaves, including decreased K and unchanged Na concentrations, were disrupted in *sgn3–3 myb36-2*, which exhibited increases in both K and Na (Reyt et al., 2021). The salt hypersensitivity in *esbl* mutant plants described above was apparent under low-humidity conditions (Wang et al., 2022). These results highlight the importance of Casparian strips in elemental homeostasis, especially when the transpiration rate is high. Our observations suggest that Casparian strips are particularly important for limiting the translocation of excess B to avoid toxicity in actively transpiring plants.

We investigated boric acid flux using a genetically encoded biosensor. In a previous study using *proUBQ10:NIP5;1 5*′*UTR : Eluc-PEST*, we successfully visualized the process of B export from roots within a few hours, dependent on an ectopically expressed borate exporter (Fukuda et al., 2018). In this study, we utilized this sensor under the control of the universal *UBQ10* promoter and also the stele-specific *CIF2* promoter (**Supplementary Figure 3**, **Figure 5**). Because changes in luminescence require time for the turnover of Eluc-PEST proteins, a time course of such changes includes a time lag after the actual change in boric acid concentration within the cell. Nevertheless, our system was able to visualize boric acid transport in local regions compared to the direct measurement of B concentrations. When boric acid was applied at a sufficient or slightly excessive concentration (300 µM), boric acid flux into the stele of root tip region was rapid and did not differ between the WT and *sgn3* and *sgn4* mutants (**Figures 5C, D**). In the mature root region, the boric acid flux into the outer cell layers was also rapid and did not differ between the WT and the *sgn3-3* mutant (**Supplementary Figure 3**), while that into the stele was slow in the WT and faster in the *sgn3* and *sgn4* mutant plants (**Figures 5E, F**). These results are consistent with the hypothesis that apoplastic diffusion from the root surface to the stele is prevented in a mature region with functional Casparian strips but not in a root tip region without Casparian strip development.

In our ^10^B tracer experiment (**Figure 4**) and biosensor imaging (**Figure 5**), plants grown under low-B conditions were supplied with high concentrations of B (1 µM to 300 µM and 0.5 µM to 300 µM, respectively). These large changes of B supply were required for detection of B flows in our experimental system. As low B supply increases the abundance of B transport proteins, such as boric acid channel NIP5;1 and borate exporter BOR1 (Takano et al., 2005; Takano et al., 2006), boric acid transport into the stele might accelerate under such conditions. However, defects of the Casparian strips also impacted plant growth and B accumulation when B was continuously supplied at high concentrations (**Figures 2**, **3**, **Supplementary Figures 1**, **2**). Considering these results, Casparian strips can be reasonably considered to play a role in the prevention of B translocation under high-B conditions irrespective of the plant’s previous B status.

We also observed a significant role of the Casparian strip in plant growth under low-B conditions. Specifically, *sgn3* and *sgn4* plants showed significant reductions in shoot growth in hydroponic culture containing 0.1 µM B (**Supplementary Figure 1**). In this condition, the B concentrations in the mutant shoots and Col-0 were comparable (**Figure 3**). In two independent ionome analyses with low (0.3 µM) B supply, a significant difference in B concentrations was found in Experiment 1 (*sgn3–4* and *rbohf* showed lower B concentrations than Col-0), but not in Experiment 2 (**Supplementary Figure 2**). Therefore, the contribution of Casparian strips to B transport under low-B conditions was unclear and varied between experiments. Theoretically, Casparian strips play a role in maintaining the concentrations of elements in the apoplasm of the root stele by preventing backflow to the outer layers of the root. Reduced accumulation of K in the shoots of *sgn3* and *sgn4* mutants (Pfister et al., 2014, **Supplementary Figure 2**) may be due to such backflow. To elucidate the role of Casparian strips in B distribution under low-B conditions, further research is required. Modification of the boric acid biosensor using fluorescence reporters (Fukuda et al., 2018) to measure B concentrations in each cell will help.

In conclusion, our findings clarify the physiological significance of the Casparian strip in preventing the apoplastic diffusion of excess boric acid into the stele. Excess B and salinity, and in many cases a combination of these stresses, are major agricultural problems that decrease crop yields in arid and semi-arid areas (Nable et al., 1997; Landi et al., 2019). Our study will be a foundation for the future breeding of crop plants in such regions.

## Data availability statement

The original contributions presented in the study are included in the article/**Supplementary Material**. Further inquiries can be directed to the corresponding author.

## Author contributions

KM, NG, and JT designed the research. KM, JK and SW performed experiments. NG produced materials. KM and JT wrote the paper. All authors contributed to the article and approved the submitted version.
