# Supplementary material for: Casparian strips prevent apoplastic diffusion of boric acid into root steles for excess B tolerance
Source: Front Plant Sci. 2023 Dec 14;14:988419. doi: 10.3389/fpls.2023.988419 (PMC10755862; doi:10.3389/fpls.2023.988419)
Supplement: Supplementary file 1 [file DataSheet_1.pdf]

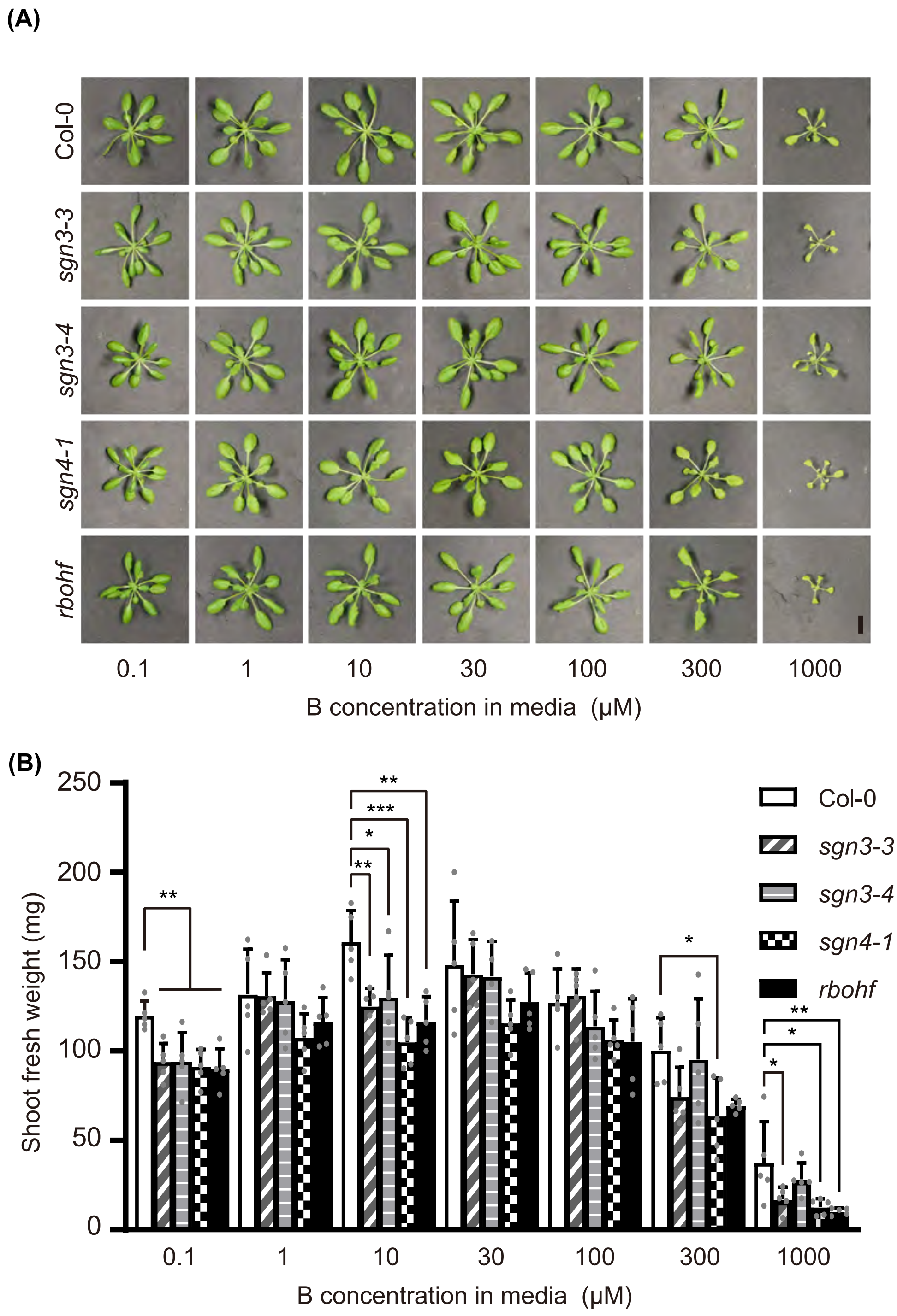

Supplementary Figure 1. Growth of Casparian strip mutants under various B concentrations in hydroponic culture. (A) Representative photograph of Col-0, *sgn3–3*, *sgn3–4*, *sgn4–1*, and *rbohf* plants grown hydroponically for 5 weeks with liquid medium containing 0.1, 1, 10, 30, 100, 300, or 1,000 μM boric acid. Scale bar = 1 cm. (B) Fresh weights of shoots from plants grown as described in (A). Data are means ± SDs (n = 4–5). Asterisks indicate significant differences between mutant plants and Col-0 (one-way ANOVA with Dunnett' s post-hoc test, $*P < 0.05$, $**P < 0.01$, $***P < 0.001$).

Supplementary Figure 2 Ionomic analysis of the Casparian strip mutants. Experiment 1.

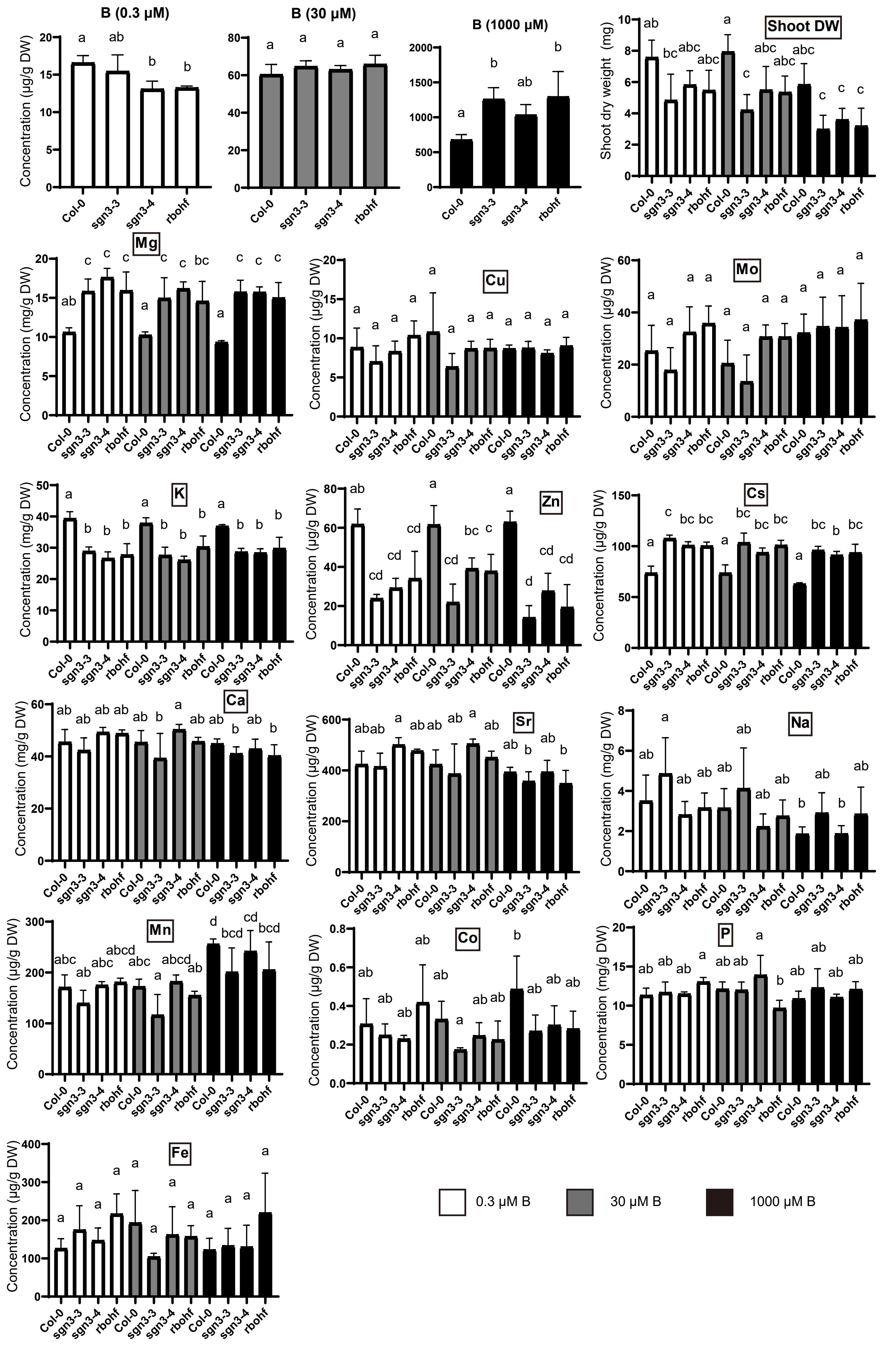

Supplementary Figure 2 Ionomic analysis of the Casparian strip mutants. Experiment 2.

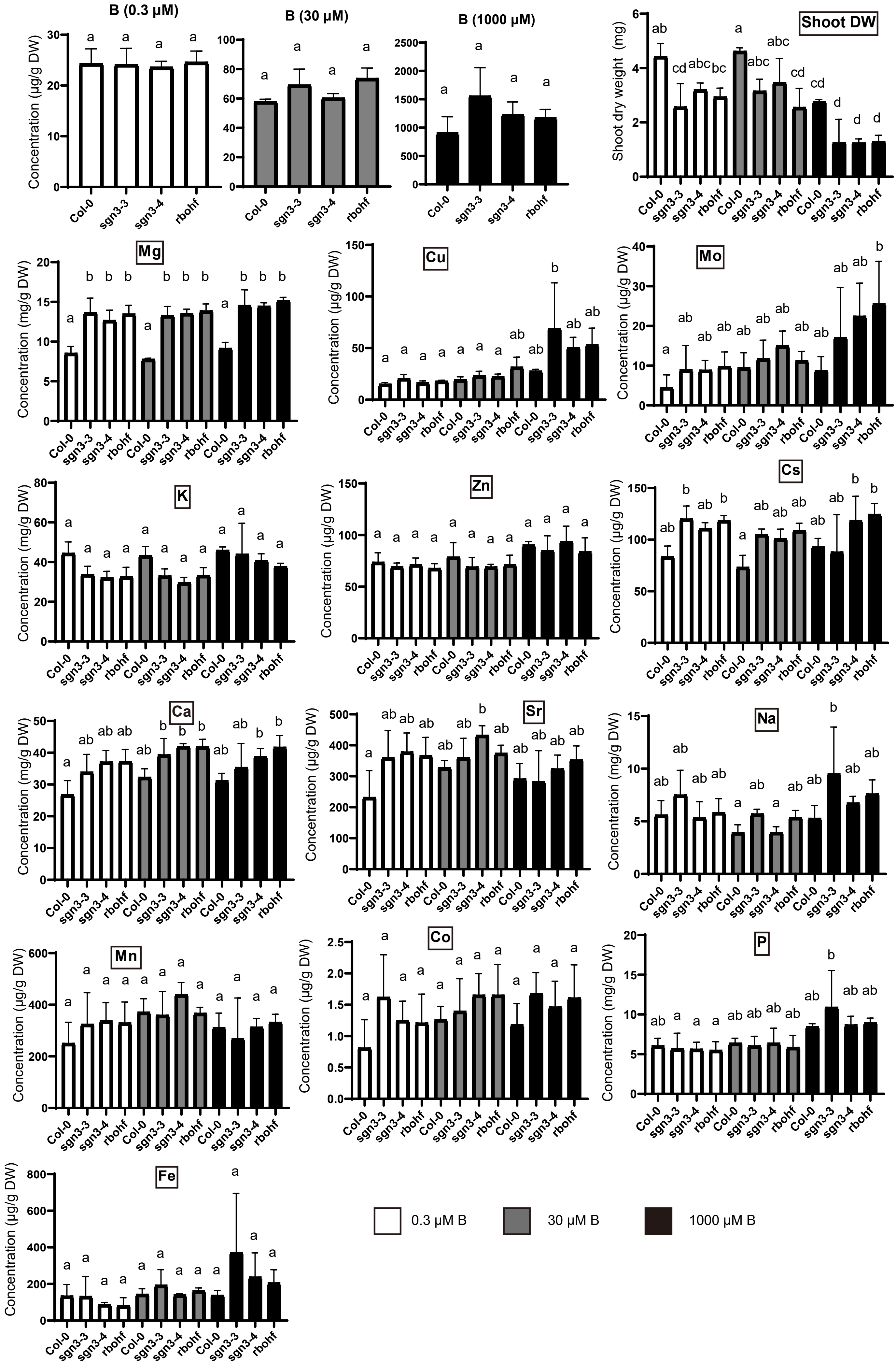

Supplementary Figure 2. Ionomic analysis of the Casparian strip mutants.

Element concentrations in the shoots of Col-0, *sgn3-3*, *sgn3-4*, *rbohf* grown hydroponically for 25 days in medium containing 0.3 μM, 30 μM or 1000 μM boric acid. The data in the 30 μM boric acid condition was already presented in the previous study (Pfister et al., 2013). The values are presented as the means ± S. D. (n = 4). Different letters indicate the significant difference by one-way ANOVA followed by Tukey' s test ($P < 0.01$).

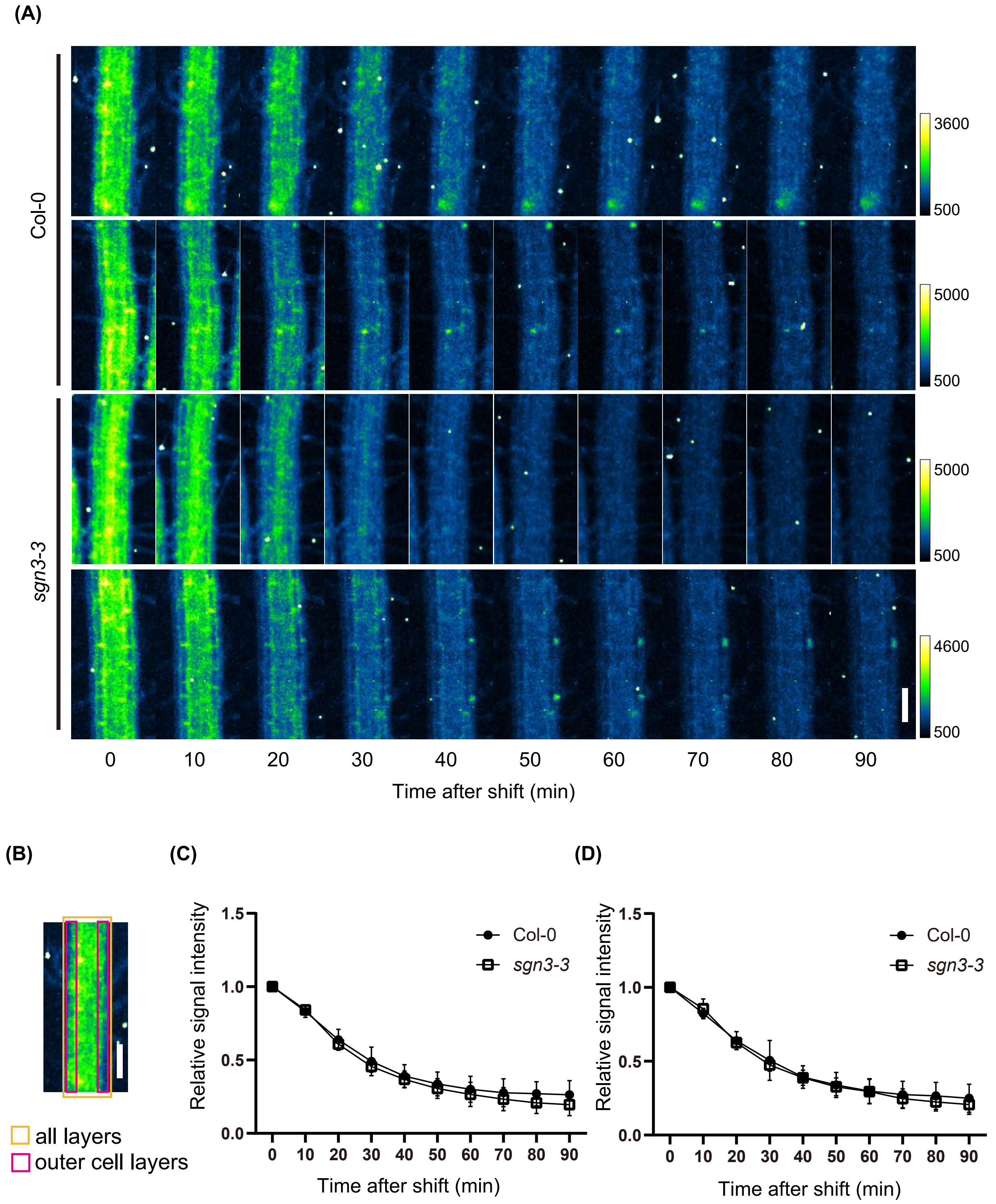
(A)
Col-0
sgn3-3
3600
500
5000
500
5000
500
4600
500
0
10
20
30
40
50
60
70
80
90
Time after shift (min)
(B)
all layers
outer cell layers
(C)
Relative signal intensity
Col-0
sgn3-3
Time after shift (min)
(D)
Relative signal intensity
Col-0
sgn3-3
Time after shift (min)

Supplementary Figure 3. Time-course analysis of boric acid flux into the root cells of Casparian strip mutants using a biosensor genetically encoded for boric acid. (A) Representative images of luminescence observed under an inverted microscope equipped with an EMCCD camera, Images were taken at 5 mm from the root tips of transgenic Col-0 or *sgn3–3* plants expressing the boric acid biosensor under the control of UBQ10 promoter after transfer to media containing 300 µM boric acid. Scale bar = 100 µm. (B) A representative image of the analyzed root region. Signals from 120 µm x 500 µm regions (yellow, all layers) and surface 20 µm x 500 µm regions (magenta, outer cell layers) were analyzed. (C-D) Time course of changes in relative signal intensity in all layers (C) and outer cell layers (D) at 5 mm from the root tips of transgenic Col-0, *sgn3–3* plants after transfer to medium containing 300 µM boric acid. Relative signal intensity was calculated by dividing each signal by the initial intensity for each plant. Data are means ± SDs (n = 4 (C), 8 (D)). "Time after shift" represents the time since exposure (10 min) began. No significant difference between Col-0 and *sgn3-3* was detected by Student' s *t*-test ($P>0.05$).

Pfister, A., Barberon, M., Alassimone, J., Kalmbach, L., Lee, Y., Vermeer, J. E. M., et al. (2014). A receptor-like kinase mutant with absent endodermal diffusion barrier displays selective nutrient homeostasis defects. Elife 3, e03115. doi: 10.7554/eLife.03115.
